# Infantile Peanut Introduction and Peanut Allergy in Regions With a Low Prevalence of Peanut Allergy: The Japan Environment and Children’s Study (JECS)

**DOI:** 10.2188/jea.JE20230210

**Published:** 2024-07-05

**Authors:** Reiji Kojima, Ryoji Shinohara, Megumi Kushima, Hideki Yui, Sanae Otawa, Sayaka Horiuchi, Kunio Miyake, Hiroshi Yokomichi, Yuka Akiyama, Tadao Ooka, Zentaro Yamagata, the Japan Environment and Children’s Study Group

**Affiliations:** 1 Department of Health Sciences, School of Medicine, University of Yamanashi, Yamanashi, Japan; 2 Center for Birth Cohort Studies, University of Yamanashi, Yamanashi, Japan; 3 Department of Epidemiology and Environmental Medicine, School of Medicine, University of Yamanashi, Yamanashi, Japan

**Keywords:** infantile peanut introduction, peanut allergy, weaning

## Abstract

**Background:**

In regions with a high prevalence of peanut allergy (PA), there is a consensus that the introduction of peanuts in early infancy is preventive against the development of PA. However, few studies have investigated whether the introduction of peanuts to infants is associated with PA in regions with a low prevalence of PA, including Japan.

**Methods:**

We used data from 74,240 mother–child pairs who participated in the Japan Environment and Children’s Study, a prospective birth cohort recruited between January 2011 and March 2014. A logistic regression model was used to analyze the association between infantile peanut introduction and PA at the age of 4 years with non-infantile peanut introduction as the reference group, adjusted for potential confounders.

**Results:**

The percentage of infantile peanut introduction was 4.9% (*n* = 3,294), and 286 (0.4%) participants had allergic symptoms to peanuts at 4 years of age. Of all participants, 129 (0.2%) had PA at 4 years of age, which was defined as allergic symptoms and sensitization to peanuts. Those with infantile peanut introduction had a lower prevalence of PA than those without infantile peanut introduction, although this did not reach statistical significance (adjusted odds ratio 0.53; 95% confidence interval, 0.17–1.68). Sensitivity analysis using IgE-mediated symptoms caused by peanuts as the outcome showed a similar result in relation to infantile peanut introduction.

**Conclusion:**

In countries with a low prevalence of PA, the effect of infantile peanut introduction on PA prevention was unclear.

## INTRODUCTION

In Western countries, peanut allergy (PA) was reported to be increasing, with a prevalence of 1.4% to 3.0% in children, and often causes anaphylaxis.^1^^,^^2^ However, in Asia, including Japan, the prevalence of PA is less than 1%,^3^^,^^4^ which is lower than that in Western countries.^5^^,^^6^ Until around 2000, United States guidelines recommended that peanut introduction should be avoided until the age of 3 years to prevent peanut sensitization and the subsequent development of PA.^7^ However, an observational study, comparing PA prevalence between the United Kingdom and Israel in 2008^8^ and the Learning Early About Peanut (LEAP) study in 2015,^9^ led to a paradigm shift whereby early peanut introduction is seen as protective against the development of PA. In response, a global consensus guideline was issued by leading international allergy, immunology, and pediatric bodies.^10^ Further follow-up studies by the Enquiring About Tolerance (EAT) study^11^ and subsequent meta-analyses^12^^,^^13^ led to a consensus that the “early introduction of peanuts for the prevention of PA” is the best method to prevent the onset of PA. However, the consensus statement was limited to “areas with a high prevalence of PA”.^10^ A commentary on the consensus by The Japanese Society for Allergology stated that “whether peanuts should be actively consumed during the early weaning period in Japan remains an important issue for future study”.^14^

The prevalence of PA is lower in Japan than in Western countries. Although studies of the prevalence of PA in the Japanese population are limited, Kusunoki et al reported that it was approximately 0.3% in a general population of 13,215 participants aged 7–15 years.^15^ Regarding the timing of peanut introduction, the Japanese Guidelines for Food Allergy 2021 only refer to the consensus communication and do not explicitly recommend early peanut introduction.^16^ In addition, the Japanese Guidelines for Feeding and Weaning 2019 state that 5–6 months of age is appropriate for the start of weaning because no evidence shows that delaying the start of weaning or intake of certain foods has a preventive effect on the development of food allergy.^17^ However, there is no indication when peanuts should be introduced to infants. Furthermore, the Japanese Consumer Affairs Agency issued a warning in 2021 that peanuts should not be given to children until they are 5 years old to prevent choking and aspiration of peanuts,^18^ which may have an impact on the timing of peanut introduction. However, to date, no study has reported the distribution of the timing of peanut introduction to the Japanese population. Studies on the association between early peanut introduction and the development of PA in regions with a low prevalence of PA, such as Asia, are scarce. In a study of 1,152 children in the general population in Singapore, 88.7% were introduced to peanuts at the age of 10 months or later, the prevalence of PA was 0.2% at 4 years of age, and the timing of introduction of allergenic foods was not associated with food allergy.^6^ The authors reported a low prevalence of PA despite the large number of participants with late peanut introduction, and stated in the limitations that the number of PA cases was too small to conduct a multivariable analysis of the association between the time of peanut introduction and development of PA.^6^ Recently, a randomized controlled trial (RCT) of 163 Japanese infants with atopic dermatitis reported that the early introduction of small amounts of multiple allergenic foods (egg yolk, milk, wheat, soy, buckwheat, and peanut) was protective against food allergy at 18 months of age.^19^ In that study, a statistical analysis could not be performed for PA because of the small sample size.^19^

No large-scale studies have thus been conducted on PA prevalence in the Japanese population, and whether the introduction of peanuts during infancy has a preventive effect against PA in regions with a low PA prevalence, such as Asia, remains unclear. Therefore, the purpose of this study was to determine the prevalence of PA and the potential preventive effect of peanut introduction during infancy on PA in a large birth cohort of Japanese subjects.

## METHODS

### Study design and participants

The Japan Environment and Children’s Study (JECS) is an ongoing, nationwide, prospective birth cohort described in other reports.^20^^,^^21^ Briefly, between January 2011 and March 2014, more than 100,000 pregnant women were recruited and enrolled at 15 Regional Centres covering a wide area of Japan. Participants were asked to complete questionnaires twice during pregnancy and then every 6 months from 6 months postpartum. The JECS protocol was reviewed and approved by the Ministry of the Environment’s Institutional Review Board on Epidemiological Studies (IRB number: 100910001) and by the Ethics Committees of all participating institutions. All participants gave written informed consent.

This study was based on the JECS datasets jecs-ta-20190930 (2022.11.29ver) and jecs-qa-20210401 (2023.3.16ver), which were released in October 2019 and April 2021, respectively. The JECS dataset contained 104,062 fetal records. Stillbirths and miscarriages (*n* = 3,759) and participants with missing data regarding peanut introduction at age 1 (*n* = 11,823) and PA status at age 4 (*n* = 14,240) were excluded. The final study population consisted of 74,240 pairs of mothers and children.

### Variables

#### Outcomes

The primary outcome of this study was the prevalence of PA at age 4 years. PA information was reported by caregivers using a self-administered questionnaire; PA was defined with reference to previous studies,^6^ as those who met the following three criteria: i) caregiver-reported allergic reactions in their children; ii) complete or partial peanut avoidance at age 4 years; and iii) abnormal blood or skin test results to peanut in the clinic or hospital as reported by caregivers. Peanut sensitization was defined as those who met the second and third criteria above.

Immunological assessment by blood test was used as a sub-outcome. Blood samples were collected from approximately 5% of the participants in JECS (JECS sub-cohort study)^22^ and the immunological assessment was based on Ara h 2 immunoglobulin (Ig)E and IgG4 levels at ages 2 and 4 years. Ara h 2-specific IgE and IgG4 in serum were determined using densely carboxylated protein microarrays and expressed as binding units per volume (BUe/ml and BUg4/mL), which has a strong correlation with the allergen-specific IgE values measured using the UniCAP system.^23^^–^^25^ Samples with undetectable Ara h 2-specific IgE and IgG4 were allocated half the limit of detection for statistical analyses.

#### Exposure

The main exposure in the study was infantile peanut induction. At age 1 year, caregivers were asked in the questionnaire when their child started eating peanuts, with “before or at 6 months”, “at 7–8 months”, “at 9–10 months”, “at 11–12 months”, “at or after 13 months”, and “has not eaten peanuts” as response options. We defined peanut introduction before 12 months as infantile peanut induction and all other responses as non-infantile peanut induction.

### Statistical analyses

To examine the association between infantile peanut introduction and PA at age 4 years, a logistic regression model was used with non-infantile peanut introduction as the reference group. The mother’s history of allergy, passive smoking during pregnancy, mother’s peanut intake during pregnancy, annual household income, mode of delivery, sex of infant, older siblings, and infantile eczema were selected from previous studies^5^^,^^6^^,^^15^^,^^26^ and included as covariates in the logistic model. Household peanut consumption correlated with environmental peanut exposure and the potential for percutaneous peanut sensitization.^27^ The present multivariable analysis included maternal peanut consumption frequency as a surrogate indicator of household peanut consumption. For eczema in infancy, a stratified analysis was performed to assess the effect of peanut introduction in infancy in a high-risk population. As a sensitivity analysis, the association between peanut sensitization as an outcome and infantile peanut induction was analyzed using a logistic regression model. For Ara h 2 IgE and IgG4 levels, median values per infantile peanut introduction were presented and a nonparametric test, Wilcoxon’s rank sum test, was performed without adjustment. Statistical analysis was performed using SAS version 9.4 (SAS Institute, Cary, NC, USA). A *P*-value <0.05 was considered statistically significant.

## RESULTS

The characteristics of the participants are shown in Table 1. Mothers with a history of allergy accounted for 48.5% of participant pairs, and infantile eczema was present in 4.2% of children. Peanuts were introduced before or at 6 months in 0.1%, at 7–8 months in 0.6%, at 9–10 months in 1.6%, and at 11–12 months in 2.3% of participants, for a total of 3,366 (4.6%) receiving infantile peanut introduction. In addition, 95% of participants reported that children had not yet eaten peanuts at age 1 year. Furthermore, 49.0% of 1.5-year-olds, 28.0% of 2-year-olds, and 11.6% of 3-year-olds were reported to have never eaten nuts, including peanuts (data not shown in table).

**Table 1.** Characteristics of the study participants (*n* = 74,240)

	Number	(%)
Maternal history of allergy	
Yes	35,841	(48.5)
No	38,057	(51.5)
Maternal age at pregnancy, years	
<25	8,114	(10.9)
≥25 and <30	21,356	(28.8)
≥30 and <35	26,294	(35.4)
≥35	18,476	(24.9)
Household income, million JPY/year	
<4	26,353	(35.5)
≥4 to <6	35,045	(47.2)
≥6	7,873	(10.6)
Unknown	4,969	(6.7)
Mode of delivery	
Caesarean section	14,508	(19.6)
Vaginal	59,417	(80.4)
Birth weight, g	
<2,500	6,621	(8.9)
≥2,500	67,448	(91.1)
Child’s sex	
Male	37,974	(51.2)
Female	36,266	(48.9)
Older siblings	
Yes	39,456	(53.4)
No	34,442	(46.6)
Duration of exclusive breast-feeding	
<6 months	44,215	(59.6)
At least 6 months	30,025	(40.4)
Passive smoking during pregnancy	
Yes	14,813	(20.2)
No	58,491	(79.8)
Doctor diagnosed allergy	
Infantile eczema	3,086	(4.2)
Mother’s peanut intake during pregnancy	
At least once a week	3,688	(5.1)
1–3 times a month	13,753	(19.0)
Less than once a month	55,095	(76.0)
Peanut introduction status at age 1 year	
≤6 months	79	(0.1)
7–8 months	440	(0.6)
9–10 months	1,154	(1.6)
11–12 months	1,693	(2.3)
Intake after 13 months	367	(0.5)
Not ingested	70,507	(95.0)

JPY, Japanese yen.

Table 2 shows the PA status at age 4 years. Of note, 8.7% of participants reported that they had never eaten peanuts; 89.0% had eaten peanuts without problems; 1.6% had partially removed them from the diet, and 0.7% had previously eaten them but were now removing them from the diet completely. There were 286 respondents (0.4%) who had allergic symptoms after consuming peanuts at age 4 years, with skin symptoms being the most common symptom (276 respondents). No respondents had shock symptoms. There were 241 (0.4%) participants with peanut sensitization and 129 (0.2%) participants with PA, who had both allergic symptoms, sensitization, and subsequently removed peanuts from their diet.

**Table 2.** Peanut allergy status at 4 years of age (*n* = 74,240)

	Number	(%)
Consumption status of peanut intake at age 4 years		
No avoidance (eating normally)	66,062	(89.0)
Has never eaten	6,428	(8.7)
Partial avoidance	1,212	(1.6)
Has eaten before; however, now complete avoidance	538	(0.7)
Symptom for peanut at age 4 years^a^		
Any symptoms	286	(0.4)
Skin symptoms	276	(0.4)
Nasal symptoms	60	(0.1)
Respiratory symptoms	50	(0.1)
Gastrointestinal symptoms	63	(0.1)
Shock symptoms	0	(0.0)
Peanut sensitization^a,b^	241	(0.4)
Peanut allergy^a,c^	129	(0.2)

^a^“Has never eaten” was excluded and 67,812 participants were used as the denominator.

^b^Peanut sensitization met the following two criteria: i) complete or partial peanut avoidance at age 4 years; and ii) abnormal blood or skin test results in the clinic or hospital.

^c^Peanut allergy met the following three criteria: i) parent-reported allergic reactions in their children; ii) complete or partial peanut avoidance at age 4 years; and iii) abnormal blood or skin test results in the clinic or hospital.

The association between infantile peanut introduction and PA is shown in Table 3. The point estimate was in the direction of the prevention of PA, with a lower prevalence of PA for those receiving infantile peanut introduction than those not receiving infantile peanut introduction, although this did not reach statistical significance (adjusted odds ratio [aOR] 0.53; 95% confidence interval [CI], 0.17–1.68; adjusted risk difference [RD] −0.0009; 95% CI, −0.0016 to 0.0013). To examine the effect of peanut introduction on a high-risk population, an analysis was performed stratified by infantile eczema (Table 3). A multivariable analysis could not be performed because none of the children with infantile eczema who were introduced to peanuts during infancy had PA at age 4 years. However, there was a significant risk difference among participants with infantile eczema (RD −0.0085; 95% CI, −0.0121 to −0.0050). For participants without infantile eczema, the results were similar to the overall results (aOR 0.65; 95% CI, 0.20–2.05). A sensitivity analysis using the likely IgE-mediated symptoms caused by peanuts as the outcome, which excluded “sensitization” from the current study’s definition of PA, showed a similar result in relation to infantile peanut introduction (aOR 0.66; 95% CI, 0.35–1.25; eTable 1). Another sensitivity analysis using peanut sensitization as an outcome showed a significant association between infantile peanut induction and peanut sensitization (aOR 0.28; 95% CI, 0.09–0.88; eTable 2).

**Table 3.** Odds ratios of peanut allergy in relation to infantile peanut introduction

	Number/Total number	(%)	Crude OR or RD	95% CI	Adjusted OR or RD^a^	95% CI
Total						
Infantile peanut introduction						
Yes	3/3,294	0.09	0.47	0.15–1.47	0.53	0.17–1.68
No	126/64,518	0.20	ref		ref	
Risk difference			−0.0010	−0.0021 to 0.00	−0.0009	−0.0016 to 0.0013

Without infantile eczema						
Infantile peanut introduction						
Yes	3/3,179	0.09	0.56	0.18–1.77	0.65	0.20–2.05
No	104/61,936	0.17	ref		ref	
Risk difference			−0.0007	−0.0019 to 0.0004	−0.0006	−0.0013 to 0.0018
With infantile eczema						
Infantile peanut introduction						
Yes	0/115	0.00	N/A		N/A	
No	22/2,582	0.85				
Risk difference			−0.0085	−0.0121 to −0.0050	N/A	

CI, confidence interval; N/A, not applicable; OR, odds ratio; RD, risk difference.

^a^Adjusted for maternal history of allergy, passive smoking, maternal peanut intake during pregnancy, house income, mode of delivery, child sex, older siblings, and infantile eczema (unless stratified by the factor).

The median values of IgE and IgG4 for Ara h 2 by infantile peanut introduction are shown in Table 4. Approximately 98% of Ara h 2 specific IgE was below the detection limit at 2 and 4 years of age, with no significant differences. There were also no significant differences in Ara h 2 specific IgG4 between the groups at 2 and 4 years of age.

**Table 4.** Immunologic status at 2 and 4 years of age in relation to infantile peanut introduction

	2 years old					4 years old				
	
	Total number	Median (IQR)	BLD number	(%)	*P*-value^a^	Total number	Median (IQR)	BLD number	(%)	*P*-value^a^
Ara h 2 specific IgE (BUe/mL)										
Infantile peanut introduction										
Yes	204	0.005 (0.005–0.005)	201	(98.5)	0.71	187	0.005 (0.005–0.005)	183	(97.8)	0.57
No	4,033	0.005 (0.005–0.005)	3,985	(98.8)		3,766	0.005 (0.005–0.005)	3,707	(98.4)	
Ara h 2 specific IgG4 (BUg4/mL)										
Infantile peanut introduction										
Yes	204	60.9 (58.8–63.5)	0	(0.0)	0.07	187	51.8 (50.1–56.0)	44	(22.7)	0.36
No	4,033	60.6 (58.3–62.9)	0	(0.0)		3,767	51.9 (50.2–54.6)	855	(21.7)	

BLD, below the detection limit; IQR, interquartile range.

^a^The Wilcoxon rank sum test was used for the analysis.

## DISCUSSION

Analysis of a large Japanese birth cohort revealed a 0.2% prevalence of PA at age 4 years. Those with infantile peanut introduction had a lower prevalence of PA than those without infantile peanut introduction, although this did not reach statistical significance. Whether peanut induction in infancy was protective against PA in the participants in our study was unclear.

The prevalence of PA in the present study was 0.2%, similar to a previous study of the Japanese general population aged 7–15 years, which reported a prevalence of approximately 0.3%.^15^ A study from Singapore reported a prevalence of PA of 0.2% in 4-year-olds,^6^ which was similar to that in the present study. Why the prevalence of PA is lower in Asian countries compared with Western countries is not fully understood.^5^ However, there are differences in peanut consumption and processing, as well as environmental microbial exposure between Asian and Western countries, which might explain the differences in PA prevalence.^28^ An Australian immigration study suggested that the incidence of PA was higher in Australian-born Asian children compared with Australian-born Caucasians, suggesting gene–environment interactions.^28^

The LEAP study showed early peanut introduction had a high preventive effect on the development of PA in a high-risk population for PA in the United Kingdom.^9^ The subsequent EAT study investigated the early introduction of multiple foods, including peanuts, to a general population of infants in the United Kingdom and found a significant preventive effect for PA in the per-protocol analysis, but not in the intention-to-treat analysis.^11^ The results of the current study were not consistent with these studies because the low prevalence of PA in Asia might have insufficient statistical power.^5^ In addition, differences in peanut processing methods during peanut introduction and consumption between Asian and Western countries may have contributed to the lack of significant preventive effects, but further research is needed. A study of 1,152 individuals in the general population of Singapore by Tham et al reported that 88.7% of children were introduced to peanuts at age 10 months or later, and that 0.2% of children had PA at age 4 years.^6^ Thus, Singapore has a low prevalence of PA despite the large number of participants with late peanut introduction.^6^ Our results support those of Tham et al.^6^ Furthermore, our multivariable analysis showed no clear preventive effect of infantile peanut introduction on the development of PA. Although our study was conducted in the general population, stratified analysis by infantile eczema, a risk factor for food allergy, was performed to examine the effect of peanut introduction on a high-risk population. As a result, no children with infantile eczema who were introduced to peanuts in infancy had PA at 4 years of age; therefore, the multivariable analysis could not be performed. Although significant RD was found in participants with infantile eczema, the sample size was small and further research is needed. Sensitivity analysis showed a significant association between induction and peanut sensitization. Caution should be taken when interpreting these results as a preventive effect of induction of PA. This is because the presence of sensitization does not necessarily correspond to allergic symptoms, and in our study, it was unclear whether this was a result of the Ara h 2-specific IgE value, which has high diagnostic accuracy. There were also no significant differences between groups at age 2 or 4 years by immunological assessment with serum IgE and IgG4 for Ara h 2, which was examined in a subset of participants.

Regarding the timing of peanut introduction, the results of this study showed that 95% of children at age 1 year and 8.7% by age 4 years had never eaten peanuts. This was similar to the Singapore report where 89% of participants were introduced to peanuts at the age of 10 months or later.^6^^,^^29^ On whether infantile peanut introduction should be added to the Japanese Guidelines for Feeding and Weaning, our study provided no evidence that peanuts should be introduced in infancy to prevent PA, because our study showed no clear PA preventive effect of introducing peanuts in infancy. Therefore, these findings suggest that in Japan, it is not the situation to recommend the introduction of peanuts during infancy to prevent the development of PA.

Revisions to weaning guidelines have had a significant impact on infant weaning practices. In Australia, weaning guidelines were revised in 2016, recommending peanut introduction for up to 12 months to prevent allergy.^26^ As a result, the percentage of infants with early peanut introduction before 12 months has increased (85.6% in 2018–2019 vs 21.6% in 2007–2011).^26^^,^^30^ However, no significant decrease in the prevalence of PA at 4 years of age was observed in the whole population so far.^26^ Moreover, studies have reported an increase in hospitalizations for food allergy-induced anaphylaxis in infants since 2016,^31^ and an increase in peanut food protein-induced enterocolitis syndrome,^32^ although these events are rare. Studies have also reported an increase in peanut aspiration in infants as a possible consequence of allergy prevention by early peanut introduction.^33^ Thus, even in regions with a high prevalence of PA, benefit and harm have been reported after the early introduction of peanuts following the revised guidelines. In regions with a low prevalence of PA, such as Japan and other Asian countries, the Western recommendation to introduce peanuts in infancy with the expectation of a preventive effect against PA should be taken with caution.

The strength of this study was the high generalizability of the results to a Japanese population.^21^ In addition, the longitudinal design of the study prevents causal reversal. However, this study had several limitations. First, PA in our study was obtained from reports from caregivers and was not confirmed by oral food challenge, which is the gold standard for food allergy diagnosis. However, the combined outcome of likely IgE-mediated symptoms and peanut sensitization allows for comparisons with previous studies.^6^ Second, the timing of peanut introduction was reported by caregivers and could be misclassified. If peanut introduction was initiated in infancy and there had been a reaction, the peanut introduction would be discontinued and remain an elimination, and the response on the questionnaire might be incorrect as “never eaten”. Therefore, the results of this study may have overestimated the preventive effect. There was also the possibility of misclassification due to the peanut ingredients in the food and the infant ingesting them without the caregivers being aware of it. In such cases, the preventive effect of infantile peanut introduction would be underestimated. In addition, the amount of peanut introduction was not investigated, although the timing of peanut introduction was. This might explain why the effect of peanut introduction during infancy on the prevention of PA was not consistent with previous studies.^9^^,^^11^ This finding might be related to differences in the amount of peanut exposure, but this could not be examined in the present study. While the EAT study investigated early introduction at 3–6 months of age,^11^ the present study could not examine early peanut introduction, as only 0.1% of cases were introduced to peanuts before 6 months of age. Future research on the introduction of peanuts in early infancy is needed. Finally, because this was an observational study and not an RCT, there were confounding factors between peanut induction and PA. Although we adjusted for known confounders^5^^,^^6^^,^^15^^,^^26^ in this study, including the mother’s history of allergies, residual confounding factors could not be ruled out. Ideally, an RCT of peanut induction should be performed; however, as the present results show, this is not feasible because of the low prevalence of PA in the Japanese population, which requires a larger sample size.

In conclusion, a large birth cohort in Japan showed that the prevalence of PA at age 4 years was 0.2%. Those with infantile peanut introduction had a lower prevalence of PA than those without infantile peanut introduction, although this did not reach statistical significance, indicating that infantile peanut introduction has no clear PA-preventive effect. The consensus stated that “early peanut introduction is recommended for allergy prevention in areas with a high prevalence of PA”; however, in countries with a low prevalence of PA, the effect of infantile peanut introduction for PA prevention is unclear.

## SUPPLEMENTARY MATERIAL

eTable 1. Odds ratios of peanut reaction in relation to infantile peanut introduction

eTable 2. Odds ratios of peanut sensitization in relation to infantile peanut introduction
