# Supplementary material for: Infantile Peanut Introduction and Peanut Allergy in Regions With a Low Prevalence of Peanut Allergy: The Japan Environment and Children’s Study (JECS)
Source: J Epidemiol. 2024 Jul 5;34(7):324–30. doi: 10.2188/jea.JE20230210 (PMC11167260; doi:10.2188/jea.JE20230210)
Supplement: Supplementary file 1 [file je-34-324-s001.pdf]

eTable 1. Odds ratios of peanut reaction in relation to infantile peanut introduction

	Number/Total Number	(%)	Crude OR	95％CI	Adjusted OR [a]	95％CI
Total						
Infantile peanut introduction						
Yes	10/3,294	0.3	0.63	0.33–1.18	0.66	0.35–1.25
No	312/64,518	0.48	ref		ref	
Without infantile eczema						
Infantile peanut introduction						
Yes	9/3,179	0.28	0.63	0.33–1.23	0.67	0.34–1.30
No	277/61,936	0.45	ref		ref	
With infantile eczema						
Infantile peanut introduction						
Yes	1/115	0.87	0.64	0.09–4.70	0.59	0.08–4.42
No	35/2,582	1.36				

CI, confidence interval; N/A, not applicable; OR, odds ratio; RD, risk difference.

[a] Adjusted for maternal history of allergy, passive smoking, maternal peanut intake during pregnancy, house income, mode of delivery, child sex, older siblings, and infantile eczema (unless stratified by the factor).

eTable 2. Odds ratios of peanut sensitization in relation to infantile peanut introduction

	Number/Total Number	(%)	Crude OR	95％CI	Adjusted OR [a]	95％CI
Total						
Infantile peanut introduction						
Yes	3/3,294	0.09	0.25	0.08–0.77	0.28	0.09–0.88
No	238/64,518	0.37	ref		ref	
Without infantile eczema						
Infantile peanut introduction						
Yes	3/3,179	0.09	0.31	0.10–0.97	0.35	0.11–1.10
No	188/61,936	0.3	ref		ref	
With infantile eczema						
Infantile peanut introduction						
Yes	0/115	0.00	N/A		N/A	
No	50/2,582	1.94				

CI, confidence interval; N/A, not applicable; OR, odds ratio; RD, risk difference.

[a] Adjusted for maternal history of allergy, passive smoking, maternal peanut intake during pregnancy, house income, mode of delivery, child sex, older siblings, and infantile eczema (unless stratified by the factor).
